# Association of Social Network Use With Increased Anxiety Related to the COVID-19 Pandemic in Anesthesiology, Intensive Care, and Emergency Medicine Teams: Cross-Sectional Web-Based Survey Study

**DOI:** 10.2196/23153

**Published:** 2020-09-24

**Authors:** Thomas Clavier, Benjamin Popoff, Jean Selim, Marion Beuzelin, Melanie Roussel, Vincent Compere, Benoit Veber, Emmanuel Besnier

**Affiliations:** 1 Department of Anesthesiology and Critical Care Rouen University Hospital Rouen France; 2 Department of Critical Care Dieppe General Hospital Dieppe France; 3 Department of Emergency Medicine Rouen University Hospital Rouen France

**Keywords:** social network, nurse, physician, anxiety, emergency medicine, anesthesiology, critical care medicine, coronavirus disease 2019, mental health, COVID-19

## Abstract

**Background:**

Critical care teams are on the front line of managing the COVID-19 pandemic, which is stressful for members of these teams.

**Objective:**

Our objective was to assess whether the use of social networks is associated with increased anxiety related to the COVID-19 pandemic among members of critical care teams.

**Methods:**

We distributed a web-based survey to physicians, residents, registered and auxiliary nurses, and nurse anesthetists providing critical care (anesthesiology, intensive care, or emergency medicine) in several French hospitals. The survey evaluated the respondents’ use of social networks, their sources of information on COVID-19, and their levels of anxiety and information regarding COVID-19 on analog scales from 0 to 10.

**Results:**

We included 641 respondents in the final analysis; 553 (86.3%) used social networks, spending a median time of 60 minutes (IQR 30-90) per day on these networks. COVID-19–related anxiety was higher in social network users than in health care workers who did not use these networks (median 6, IQR 5-8 vs median 5, IQR 3-7) in univariate (*P*=.02) and multivariate (*P*<.001) analyses, with an average anxiety increase of 10% in social network users. Anxiety was higher among health care workers using social networks to obtain information on COVID-19 than among those using other sources (median 6, IQR 5-8 vs median 6, IQR 4-7; *P*=.04). Social network users considered that they were less informed about COVID-19 than those who did not use social networks (median 8, IQR 7-9 vs median 7, IQR 6-8; *P*<.01).

**Conclusions:**

Our results suggest that social networks contribute to increased anxiety in critical care teams. To protect their mental health, critical care professionals should consider limiting their use of these networks during the COVID-19 pandemic.

## Introduction

The emergence of SARS-CoV-2, which causes the disease COVID-19, at the end of 2019 caused a large global outbreak and represents a major public health issue. While 80% of patients appear to be mildly symptomatic or asymptomatic, about 20% develop viral pneumonia. Of these severe forms, 7% to 40% of patients progress to acute respiratory distress syndrome and may require admission to the intensive care unit (ICU) [1,2]. Although currently there are few data on the mortality of this infection, early reports suggest a high mortality rate of approximately 6% for all patients with COVID-19 and of up to 60% among patients admitted to the ICU [2,3].

Physicians and nurses providing critical care (eg, intensivists, anesthesiologists, and emergency physicians) are on the front line of management of the most severe forms of COVID-19. The viral load of SARS-CoV-2 detected in patients’ respiratory tracts is positively linked to lung disease severity; therefore, patients with COVID-19 who are admitted to the emergency department or ICU are probably the most contagious [4]. Significant risk of transmission of SARS-CoV-2 from patients to health care workers has been described [5]. It is therefore obvious that given the risk of contamination and the high mortality rate of COVID-19, this pandemic is a great source of stress for health care workers [6].

Social networks (eg, Twitter and Facebook) enable users to find information by passively viewing a message or information thread without using traditional media, while instant messaging platforms (eg, WhatsApp) enable users to communicate directly with friends or colleagues. These apps are now commonly used by health care workers in many domains, such as teaching, promotion of scientific work, contact with patients, and discussion with colleagues [7-9]. Some health care workers based in areas that are currently severely affected by the COVID-19 pandemic (ie, northern Italy, eastern France, and New York City in the United States) use these tools to communicate and share their stressful experiences. Thus, since the beginning of the COVID-19 pandemic, many testimonials have emerged on social networks of issues such as lack of ICU beds, necessity to make difficult ethical decisions, and high numbers of deaths despite optimal care. Repeatedly reading such information can be a source of anxiety for health care workers who are already in contact with patients with COVID-19 or for those who have not yet been in contact with these patients. To preserve the mental health of these health care workers, who are essential to the functioning of the emergency organizations established in affected countries, it appears to be necessary to find strategies to limit the anxiety of health care workers. It is thus crucial to understand and analyze the sources of this anxiety.

The objective of this work was to assess whether the use of social networks and instant messaging apps to obtain and exchange information on the COVID-19 pandemic is associated with increased anxiety in critical care teams.

## Methods

### Population Selection

The Ethics and Evaluation Committee for Non-Interventional Research of Rouen University Hospital approved the study (No. E2020-12). We conducted a prospective study in France using a declarative survey. The link to an open, web-based Google Forms survey with 20 items on one webpage was sent by email to medical and paramedical teams in anesthesiology, intensive care, and emergency departments throughout France via professional or personal emailing lists (including lists belonging to department heads of the Rouen University Hospital). The survey was also distributed via professional WhatsApp discussion groups to which the authors belonged. Finally, two associative or academic societies (the Association of Young Anesthesiologists and Intensivists and the French Intensive Care Society) also relayed the questionnaire; the method of dissemination was left to the discretion of the community manager of each society. In practice, the questionnaire was disseminated to both societies via Twitter. All contacted health care workers were asked to forward the survey link to their colleagues. All participants received information about the survey objectives, which were recalled in the preface of the questionnaire. By voluntarily participating in the survey after receiving adequate information on its purpose, informed consent was implied. Although it was theoretically possible to identify individual participants, no efforts were made to do this, and no plausible harm to participating individuals could arise from the study. This survey was developed according to available guidelines for self-administered surveys [10]. Responses were entered on a single webpage with one Submit button that only allowed submissions via a unique link; thus, uninvited responses were extremely unlikely. The request was sent to 172 health care workers at our home institution; however, as we were unable to determine how many health care workers the request was forwarded to at other institutions, we do not know how many health care workers received the request to participate in the survey. The survey was conducted in accordance with the Checklist for Reporting Results of Internet E-surveys (CHERRIES) [11].

The participants included in the analysis were medical health care workers (physicians and residents) or paramedical health care workers (registered nurses, auxiliary nurses, and nurse anesthetists) who were in contact with patients in a critical care sector of a French hospital: anesthesiology, ICU, emergency department, mobile emergency, critical care unit, or mixed activity. Criteria for noninclusion were other health professions, health care workers who worked in other hospital departments or in a country other than France, and professionals who had no direct interaction with patients (eg, nursing managers).

### Objectives

Our main objective was to compare the levels of anxiety related to the COVID-19 pandemic between health care workers who use social networks and those who do not use them.

The secondary objectives were to compare the levels of information related to the COVID-19 pandemic between health care workers who use social networks and those who do not use them, to compare the levels of anxiety related to the COVID-19 pandemic between health care workers who use WhatsApp professionally and those who use WhatsApp but not professionally, and to compare the levels of anxiety related to the COVID-19 pandemic between respondents who use social networks as a source of information on the COVID-19 pandemic and those who use other information sources.

### Survey Design

The survey was designed and written by TC and then reviewed, tested and validated by EB (assistant professor) and VC (full professor) before being sent. To avoid bias related to the evolution of anxiety as the pandemic progresses, the survey period lasted only one week. The survey was constructed in three parts. The Demographic Data section analyzed the participants’ region and city of practice, age, gender, type of hospital (public or private, university), department, and profession. The Use of Social Networks section analyzed personal and professional use of WhatsApp, the social networks consulted at least once a week, and the average daily time spent on social networks. Finally, the Link Between Social Networks and the COVID-19 Pandemic section analyzed the sources of information participants used to learn about the pandemic, use of WhatsApp to discuss the pandemic with colleagues, participation in a WhatsApp group dedicated solely to discussions about COVID-19, direct interaction with a patient with COVID-19, work in a hospital that was already in contact with or was expecting to be in contact with patients with COVID-19, and subjective levels of information and anxiety about the pandemic (rated on a Likert scale from 0 to 10).

For the demographic analysis, the 14 regions of practice (13 French metropolitan regions and 1 overseas territory) were grouped into two areas according to the incidence rate of COVID-19 on March 19, 2020 (the day before the survey was released): low-density areas (regions with an incidence rate below the median incidence rate in France) and high-density areas (regions with an incidence rate above the median incidence rate in France; see Table 1).

The web-based survey can be accessed on the internet in French [12]. A version of the survey translated into English is available in Multimedia Appendix 1.

**Table 1:** Clustering of French regions in low- and high-density areas according to COVID-19 incidence.

French area and region		Cases per 100,000 inhabitants (March 19, 2020)	
**COVID-19 low-density areas**			
	Pays de la Loire		6.15
	Outre-mer (Martinique, Guadeloupe, Guyane, Réunion, Mayotte)		7.80
	Nouvelle-Aquitaine		8.68
	Centre-Val de Loire		9.93
	Occitanie		10.31
	Normandie		10.44
	Bretagne		10.75
**COVID-19 high-density areas**			
	Hauts-de-France		13.27
	Auvergne-Rhône-Alpes		15.76
	Provence-Alpes-Côte d'Azur		20.59
	Bourgogne-Franche-Comté		32.45
	Île-de-France		38.24
	Corse		50.19
	Grand Est		56.04

### Statistical Analyses

In univariate analyses, chi-square tests were performed for categorical variables and Wilcoxon tests were performed for continuous variables. A multivariable analysis by linear regression was performed to model the anxiety score and adjust for confounders, including age, region density, type of department, level of information on COVID-19, and whether the department was already providing (or was going to provide) care for patients with COVID-19. The Spearman rank correlation test was used to assess the association between two variables. The analyses were conducted bilaterally, taking a significance threshold of *P*<.05. Continuous variables were described as median (IQR) and categorical variables were described as absolute numbers and percent prevalence (%). All statistical analyses were performed using R version 3.5.1 (R Foundation for Statistical Computing).

### Data Availability

The raw data supporting the conclusions of this manuscript can be made available on request by the authors to any qualified researcher.

## Results

### Demographic Characteristics

**Figure 1:**
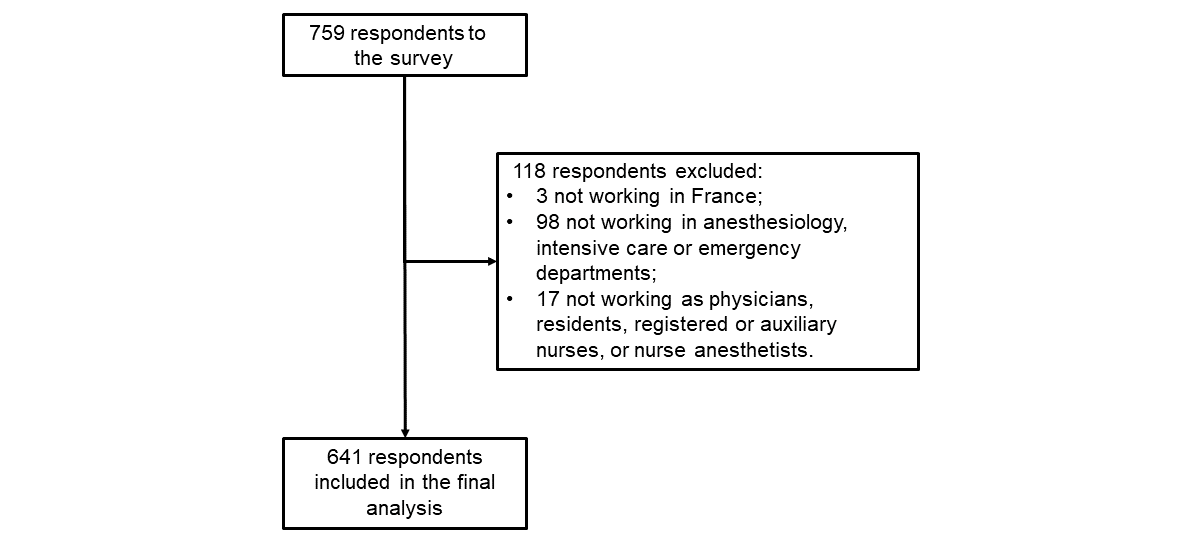
Flowchart of the selection of the study respondents.

**Figure 2:**
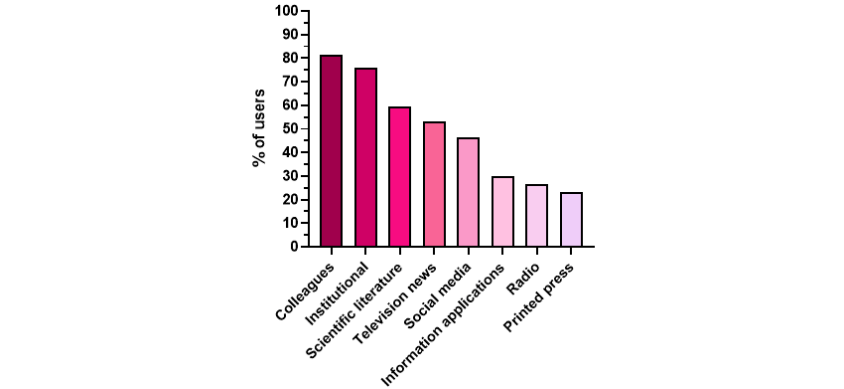
Percentages of respondents who used different media to obtain information on the COVID-19 pandemic.

The responses were compiled from March 20 to 27, 2020. A total of 759 health care workers responded to the survey; 118 respondents did not meet our inclusion criteria and were excluded from the analysis (Figure 1). Among the 641 respondents analyzed, the median age was 33 years (IQR 29-41), the sex ratio was 0.79 (282 men, 359 women), and 170 (26.5%) worked in a COVID-19 high-density area. The respondents’ main sources of information on the COVID-19 pandemic were discussion with colleagues, institutional information (from hospital management or academic societies), and scientific literature (Figure 2).

### Use of Social Networks and Anxiety

**Figure 3:**
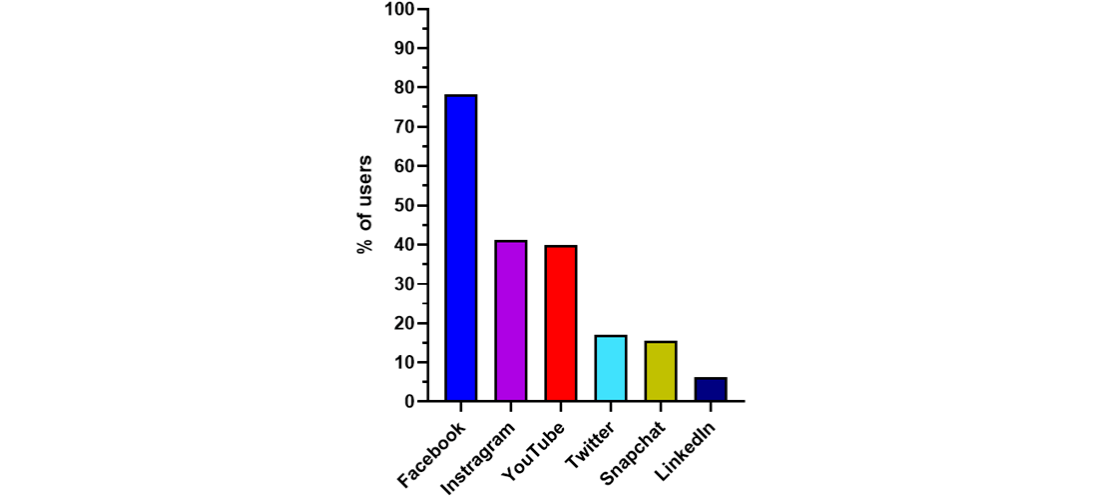
Percentages of users who consulted different social networks at least once per week (n=553).

Among the 641 respondents included in the final analysis, 553 (86.3%) were social network users, and they spent a median time of 60 minutes (IQR 30-90) per day on these networks. The social networks used by the respondents are detailed in Figure 3. In the univariate analysis, respondents who used social networks worked more in COVID-19 low-density areas and intensive care units, and they were more likely to work in a hospital that was managing patients with COVID-19 (Table 2). They also reported a higher level of anxiety regarding COVID-19 and felt less informed than respondents who did not use social networks (Table 2). In multivariate analysis adjusted for age, density of region, type of department, level of information on COVID-19, and whether the department provided care for patients with COVID-19, anxiety was significantly associated with social network use (*P*<.001), with an average increase of 1.0 anxiety point (corresponding to a 10% increase) in social network users.

Among social network users, the level of anxiety was higher among health care workers who used social networks to obtain information on the COVID-19 pandemic than among those who used other sources of information (median 6, IQR 5-8 vs median 6, IQR 4-7; *P*=.04). There was no correlation between the time spent on social networks and the level of anxiety (*r*=0.08, 95% CI 0.00 to 0.17; *P*=.05) or the level of information (*r*=0.02, 95% CI –0.06 to 0.11; *P*=.57). There was no difference in anxiety levels between health care workers who used WhatsApp to discuss COVID-19 with other professionals (median 6, IQR 5-7) and those who did not use it (median 6, IQR 4-8; *P*=.32). There was no correlation between the information level concerning the COVID-19 pandemic and the level of anxiety (*r*=–0.05, 95% CI –0.13 to 0.03; *P*=.17).

**Table 2:** Comparisons of the characteristics and use of social networks by health care workers (N=641). Percentages are expressed in relation to the number of respondents to each question.

Characteristic		Health care workers not using social networks	Health care workers using social networks	*P* value
n (%)		88 (13.7)	553 (86.3)	N/A^a^
Age (years), median (IQR)		46.0 (35.0-54.3)	32.0 (28.0-38.0)	<.001
Sex ratio (male/female)		0.87	0.96	.68
COVID-19 low-density area, n (%)		73 (83.0)	398 (72.0)	.04
**Profession, n (%)**				.41
	Physician or resident	62 (70.5)	361 (65.3)	
	Nurse (registered or auxiliary) or nurse anesthetist	26 (29.5)	192 (34.7)	
**Department, n (%)**				.02
	Anesthesiology	42 (47.7)	181 (32.7)	
	Intensive care unit	23 (26.1)	223 (40.3)	
	Emergency	6 (6.8)	55 (9.9)	
	Mobile emergency	9 (10.2)	34 (6.1)	
	Mixed	8 (9.1)	60 (10.8)	
**Hospital, n (%)**				.54
	University	52 (59.1)	325 (58.8)	
	Public	30 (34.1)	180 (32.5)	
	Private	6 (6.8)	35 (6.3)	
Working in a hospital providing care to patients with COVID-19, n (%)		65 (73.9)	474 (85.7)	<.01
Direct interaction with patients with COVID-19, n (%)		33 (37.5)	240 (43.4)	.36
Use of WhatsApp, n (%)		81 (92.0)	517 (93.5)	.78
Use of WhatsApp to discuss COVID-19 with critical care professionals, n (%)		61 (73.5)	379 (71)	.73
Use of a WhatsApp group specifically dedicated to COVID-19, n (%)		41 (50.0)	213 (40.0)	.11
Anxiety level concerning the COVID-19 pandemic (from 0 to 10), median (IQR)		5 (3-7)	6 (5-8)	.02
Information level concerning the COVID-19 pandemic (from 0 to 10), median (IQR)		8 (7-9)	7 (6-8)	<.01

^a^N/A: not applicable.

## Discussion

### Principal Results

We have shown that the use of social networks is independently associated with a higher level of anxiety among health care workers in critical care sectors. To our knowledge, we describe a link between the use of social networks and anxiety in medical teams in a pandemic context for the first time.

### Comparison With Prior Work

Several studies have shown that the COVID-19 pandemic is causing increased anxiety both in infected patients and in professionals who are in contact with these patients. Thus, rates of moderate or major anxiety ranging from 9% to 46% have been reported in health workers involved in the management of patients with of COVID-19, depending on the study [13,14]. It has been shown that in this population, age <35 years, female sex, and working in contact with patients with COVID-19 for >3 hours per day are associated with the development of an anxiety disorder due to COVID-19 [15,16]. These characteristics correspond to our population; therefore, we can assume that the population we studied is at high risk for pandemic-induced anxiety disorders.

In our study, the rate of social network users was high but consistent with data from previous studies that reported rates ranging from 50% to 88% among health care professionals [17-19]. The rate of WhatsApp users was also similar to that in a recent report, in which it was found that 98% of primary health care professionals used WhatsApp [17]. Social networks are known to be involved in the dissemination of “fake news” and rumors [20]. It has also been shown that on Facebook and YouTube, during a previous pandemic situation (Zika virus), misleading posts and videos were far more popular than those containing accurate and relevant public health information about the disease [21,22]. It is therefore very likely that many misinformation or conspiracy theories about the COVID-19 pandemic are circulating on social networks; for this reason, the World Health Organization recently warned against “trolls and conspiracy theories” about COVID-19 [23]. Thus, it is interesting to note that in our work, respondents who used social networks felt less informed than their colleagues who did not use them, and respondents who used social networks as a source of information on the COVID-19 pandemic showed a higher level of anxiety. Our data suggest that social networks are inefficient in providing quality information and that the information provided is stressful. However, even if we did not find a correlation between the respondents’ level of information concerning COVID-19 and level of anxiety in our study, it is also possible that the use of social networks to obtain information is linked to a lack of information, which itself can potentially cause anxiety. Because the design of our study did not allow us to establish a causal link, it would have been interesting to conduct interviews with a few caregivers using social networks to determine the causal link between these parameters.

The association between anxiety or depression and social network use has already been described in patients and in the general population [24,25]. However, this is the first study that specifically highlights this association in members of critical care teams. It is possible that false information (or the highly anxiety-inducing formulation of true information) may increase the anxiety experienced by health care workers; this would explain our results, at least in part. Despite the fact that time spent on social networks and anxiety score were not correlated in our study (*P*=.05), there are reports in the literature that suggest a dose-dependent relationship between time spent on social networks and anxiety [25,26]. It is known that work-related anxiety is associated with many complications in health care workers (particularly in critical care sectors), including accidents, medical errors, burnout, and secondary traumatic stress [27,28]. The COVID-19 pandemic is clearly associated with high levels of stress, sleeping disorders, and anxiety among health care professionals who interact with these patients [6,29]. It is therefore essential for these professionals to manage their mental health and to limit their sources of stress. Our results suggest that advising health care professionals to limit (or even temporarily stop) their consultation of social networks could be a way to limit this professional stress related to COVID-19.

### Limitations

Despite our interesting results, our work has several major limitations. First, because our study was observational, it is not possible to establish a causal link between the use of social networks and anxiety. This is a cross-sectional study; therefore, we did not analyze the evolution of anxiety over time. It was shown in a longitudinal study conducted over a 4-week period during the COVID-19 pandemic that the anxiety of the general population did not vary significantly [30]. However, it is questionable whether this conclusion applies to health care workers. Second, our study focused on social networks but was itself partly disseminated on Twitter, which causes selection bias and explains the high rates of social network users and young health care workers in our work. Moreover, people who responded to the Google survey may be more familiar with the use of social networks than other health care workers. However, the use of conventional email diffusion allowed us to constitute a control group with a significant number of respondents and a rate of social network users that is comparable to those described among health professionals [17-19]. Third, we estimated anxiety with a single question on a Likert scale without considering multiple psychological components (depression, anxiety, insomnia, and distress) and without using a specific neuropsychological test as previously described [6]. The study was conducted in the context of major work overloads for critical care teams in some French regions. It is therefore very likely that some health care workers did not have time to answer the questionnaire. To be able to obtain responses from high-density sectors, we therefore deliberately chose to limit ourselves to a small number of questions and to greatly simplify our assessment of anxiety. However, it has been shown that evaluation by a computerized anxiety visual analog scale is reliable and is correlated with more complex neuropsychological tests [31]. We therefore considered that our assessment of anxiety was interpretable. Fourth, the questionnaire was written, proofread, and sent out in a few days in the middle of a peak of the epidemic, and we did not have time to have it validated by sending it to experts in survey design, anxiety, or pandemic infectious disease. This may limit the relevance of some of the items analyzed (eg, the item “discussion with colleagues” does not differentiate between whether these conversations involve the use of social networks or not). Fifth, by taking data (age, sex, profession) for individual respondents and cross-referencing the information, if one has the list of names and roles of the people working in all the hospitals, there is a theoretical possibility that some participants could be identified. The anonymization of the respondents is therefore incomplete; however, no efforts were made to identify individual participants in this study. Sixth, we analyzed anxiety in health care workers; however, it is possible that because they receive superior information about the reality of the pandemic, the anxiety of health care workers may be lower than that of the general population. Recent studies have found COVID-19 pandemic anxiety syndrome rates of 29% to 45% in the general population, while a meta-analysis determined that this anxiety was only detected in 23% of health care workers [32-34]. Finally, we only collected the potentially harmful effects of social networks; however, it is also possible that, as recently suggested, use of these networks has beneficial effects that we did not record in this work, such as preservation of contact with family and friends during confinement or relaxation while watching videos [35]. It has already been shown that social networks can also be used to disseminate valid information from public health organizations [36]. In future, it would be interesting to evaluate the potential beneficial effects of these networks during the COVID-19 pandemic.

### Conclusion

Although we did not establish a direct causal link, in this paper, we have shown that the use of social networks is independently associated with increased anxiety among health care workers involved in the management of patients with severe COVID-19. To protect their mental health, critical care professionals who are already under intense stress because of the current pandemic may want to limit their use of these networks during the coming months.

## Appendix

Multimedia Appendix 1 English translation of the survey.

## Abbreviations

CHERRIES: Checklist for Reporting Results of Internet E-surveys
ICU: intensive care unit

## References

[1] Wu C, Chen X, Cai Y, Xia J, Zhou X, Xu S, Huang H, Zhang L, Zhou X, Du C, Zhang Y, Song J, Wang S, Chao Y, Yang Z, Xu J, Zhou X, Chen D, Xiong W, Xu L, Zhou F, Jiang J, Bai C, Zheng J, Song Y (2020). Risk Factors Associated With Acute Respiratory Distress Syndrome and Death in Patients With Coronavirus Disease 2019 Pneumonia in Wuhan, China. JAMA Intern Med.

[2] Yang X, Yu Y, Xu J, Shu H, Xia J, Liu H, Wu Y, Zhang L, Yu Z, Fang M, Yu T, Wang Y, Pan S, Zou X, Yuan S, Shang Y (2020). Clinical course and outcomes of critically ill patients with SARS-CoV-2 pneumonia in Wuhan, China: a single-centered, retrospective, observational study. Lancet Respir Med.

[3] Baud D, Qi X, Nielsen-Saines K, Musso D, Pomar L, Favre G (2020). Real estimates of mortality following COVID-19 infection. Lancet Infect Dis.

[4] Liu Y, Yang Y, Zhang C, Huang F, Wang F, Yuan J, Wang Z, Li J, Li J, Feng C, Zhang Z, Wang L, Peng L, Chen L, Qin Y, Zhao D, Tan S, Yin L, Xu J, Zhou C, Jiang C, Liu L (2020). Clinical and biochemical indexes from 2019-nCoV infected patients linked to viral loads and lung injury. Sci China Life Sci.

[5] Ng K, Poon BH, Kiat Puar TH, Shan Quah JL, Loh WJ, Wong YJ, Tan TY, Raghuram J (2020). COVID-19 and the Risk to Health Care Workers: A Case Report. Ann Intern Med.

[6] Lai J, Ma S, Wang Y, Cai Z, Hu J, Wei N, Wu J, Du H, Chen T, Li R, Tan H, Kang L, Yao L, Huang M, Wang H, Wang G, Liu Z, Hu S (2020). Factors Associated With Mental Health Outcomes Among Health Care Workers Exposed to Coronavirus Disease 2019. JAMA Netw Open.

[7] Clavier T, Ramen J, Dureuil B, Veber B, Hanouz J, Dupont H, Lebuffe G, Besnier E, Compere V (2019). Use of the Smartphone App WhatsApp as an E-Learning Method for Medical Residents: Multicenter Controlled Randomized Trial. JMIR mHealth uHealth.

[8] Demailly Z, Brulard G, Selim J, Compère V, Besnier E, Clavier T (2020). Gender differences in professional social media use among anaesthesia researchers. Br J Anaesth.

[9] Rolls K, Hansen M, Jackson D, Elliott D (2016). How Health Care Professionals Use Social Media to Create Virtual Communities: An Integrative Review. J Med Internet Res.

[10] Burns KEA, Duffett M, Kho ME, Meade MO, Adhikari NKJ, Sinuff T, Cook DJ, ACCADEMY Group (2008). A guide for the design and conduct of self-administered surveys of clinicians. CMAJ.

[11] Eysenbach G (2004). Improving the quality of Web surveys: the Checklist for Reporting Results of Internet E-Surveys (CHERRIES). J Med Internet Res.

[12] Réseaux sociaux et Covid-19.

[13] Chew NWS, Lee GKH, Tan BYQ, Jing M, Goh Y, Ngiam NJH, Yeo LLL, Ahmad A, Ahmed Khan F, Napolean Shanmugam G, Sharma AK, Komalkumar RN, Meenakshi PV, Shah K, Patel B, Chan BPL, Sunny S, Chandra B, Ong JJY, Paliwal PR, Wong LYH, Sagayanathan R, Chen JT, Ying Ng AY, Teoh HL, Tsivgoulis G, Ho CS, Ho RC, Sharma VK (2020). A multinational, multicentre study on the psychological outcomes and associated physical symptoms amongst healthcare workers during COVID-19 outbreak. Brain Behav Immun.

[14] Que J, Shi L, Deng J, Liu J, Zhang L, Wu S, Gong Y, Huang W, Yuan K, Yan W, Sun Y, Ran M, Bao Y, Lu L (2020). Psychological impact of the COVID-19 pandemic on healthcare workers: a cross-sectional study in China. Gen Psychiatr.

[15] Lin Y, Hu Z, Alias H, Wong LP (2020). Knowledge, Attitudes, Impact, and Anxiety Regarding COVID-19 Infection Among the Public in China. Front Public Health.

[16] Huang Y, Zhao N (2020). Generalized anxiety disorder, depressive symptoms and sleep quality during COVID-19 outbreak in China: a web-based cross-sectional survey. Psychiatry Res.

[17] Marin-Gomez FX, Garcia Cuyas F, Reig-Bolano R, Mendioroz J, Roura-Poch P, Pico-Nicolau M, Vidal-Alaball J (2018). Social Networking App Use Among Primary Health Care Professionals: Web-Based Cross-Sectional Survey. JMIR mHealth uHealth.

[18] Lefebvre C, McKinney K, Glass C, Cline D, Franasiak R, Husain I, Pariyadath M, Roberson A, McLean A, Stopyra J (2020). Social Media Usage Among Nurses: Perceptions and Practices. J Nurs Adm.

[19] Surani Z, Hirani R, Elias A, Quisenberry L, Varon J, Surani S, Surani S (2017). Social media usage among health care providers. BMC Res Notes.

[20] Grinberg N, Joseph K, Friedland L, Swire-Thompson B, Lazer D (2019). Fake news on Twitter during the 2016 U.S. presidential election. Science.

[21] Sharma M, Yadav K, Yadav N, Ferdinand KC (2017). Zika virus pandemic-analysis of Facebook as a social media health information platform. Am J Infect Control.

[22] Bora K, Das D, Barman B, Borah P (2018). Are internet videos useful sources of information during global public health emergencies? A case study of YouTube videos during the 2015-16 Zika virus pandemic. Pathog Glob Health.

[23] Depoux A, Martin S, Karafillakis E, Preet R, Wilder-Smith A, Larson H (2020). The pandemic of social media panic travels faster than the COVID-19 outbreak. J Travel Med.

[24] Shensa A, Sidani JE, Dew MA, Escobar-Viera CG, Primack BA (2018). Social Media Use and Depression and Anxiety Symptoms: A Cluster Analysis. Am J Health Behav.

[25] Lin LY, Sidani JE, Shensa A, Radovic A, Miller E, Colditz JB, Hoffman BL, Giles LM, Primack BA (2016). Association Between Social Media Use and Depression Among U.S. Young Adults. Depress Anxiety.

[26] Vannucci A, Flannery KM, Ohannessian CM (2017). Social media use and anxiety in emerging adults. J Affect Disord.

[27] Weaver MD, Vetter C, Rajaratnam SMW, O'Brien CS, Qadri S, Benca RM, Rogers AE, Leary EB, Walsh JK, Czeisler CA, Barger LK (2018). Sleep disorders, depression and anxiety are associated with adverse safety outcomes in healthcare workers: A prospective cohort study. J Sleep Res.

[28] van Mol MMC, Kompanje EJO, Benoit DD, Bakker J, Nijkamp MD (2015). The Prevalence of Compassion Fatigue and Burnout among Healthcare Professionals in Intensive Care Units: A Systematic Review. PLoS One.

[29] Xiao H, Zhang Y, Kong D, Li S, Yang N (2020). The Effects of Social Support on Sleep Quality of Medical Staff Treating Patients with Coronavirus Disease 2019 (COVID-19) in January and February 2020 in China. Med Sci Monit.

[30] Wang C, Pan R, Wan X, Tan Y, Xu L, McIntyre RS, Choo FN, Tran B, Ho R, Sharma VK, Ho C (2020). A longitudinal study on the mental health of general population during the COVID-19 epidemic in China. Brain Behav Immun.

[31] Abend R, Dan O, Maoz K, Raz S, Bar-Haim Y (2014). Reliability, validity and sensitivity of a computerized visual analog scale measuring state anxiety. J Behav Ther Exp Psychiatry.

[32] Pappa S, Ntella V, Giannakas T, Giannakoulis VG, Papoutsi E, Katsaounou P (2020). Prevalence of depression, anxiety, and insomnia among healthcare workers during the COVID-19 pandemic: A systematic review and meta-analysis. Brain Behav Immun.

[33] Wang C, Pan R, Wan X, Tan Y, Xu L, Ho CS, Ho RC (2020). Immediate Psychological Responses and Associated Factors during the Initial Stage of the 2019 Coronavirus Disease (COVID-19) Epidemic among the General Population in China. Int J Environ Res Public Health.

[34] Bäuerle A, Teufel M, Musche V, Weismüller B, Kohler H, Hetkamp M, Dörrie N, Schweda A, Skoda E (2020). Increased generalized anxiety, depression and distress during the COVID-19 pandemic: a cross-sectional study in Germany. J Public Health (Oxf).

[35] Wiederhold BK (2020). Using Social Media to Our Advantage: Alleviating Anxiety During a Pandemic. Cyberpsychol Behav Soc Netw.

[36] Daughton AR, Paul MJ (2019). Identifying Protective Health Behaviors on Twitter: Observational Study of Travel Advisories and Zika Virus. J Med Internet Res.

