# Supplementary material for: Association of Social Network Use With Increased Anxiety Related to the COVID-19 Pandemic in Anesthesiology, Intensive Care, and Emergency Medicine Teams: Cross-Sectional Web-Based Survey Study
Source: JMIR Mhealth Uhealth. 2020 Sep 24;8(9):e23153. doi: 10.2196/23153 (PMC7518883; doi:10.2196/23153)
Supplement: Multimedia Appendix 1 [file mhealth_v8i9e23153_app1.docx]

**Appendices 1**

**Social networks and Covid-19**

The objective of this study is to evaluate the impact of social networks on the information and perceptions of health professionals involved in critical care services regarding the current COVID-19 epidemic.

****Required***

**Demographic Data**

1. **In which region do you practice?**
2. **In which city do you practice?**
3. **How old are you? (in years) ***
4. **What's your gender? ***

Man

Woman

Other:…………….

1. **What type of structure do you currently work in? ***

University hospital

General Hospital

Private care institution contributing to the public service

Private Care Institution / Clinic

Other:……………

1. **What is your function?**

MD/MD-PhD

Assistant/resident

MD in private care institution

Nurse

Nurse anesthetist

Nursing assistant

Other:………………

1. **In which sector do you currently practice your main activity? ***

In intensive care unit

In anesthesiology

In a sector that combine anesthesia and intensive care

In emergency room

In mobile emergency and critical care unit

Other:……………….

**Use of the Social Networks**

1. **Do you have the WhatsApp application on your smartphone? ***

Yes

No

1. **If you have WhatsApp, do you use it for professional communications? (patient records, service organization, etc.).**

Yes

No

1. **Which of the following social networks do you consult regularly (at least once a week)? ***

Twitter

Facebook

Instagram

Snapchat

Youtube

Linkedln

I don't use social networks (or very few)

1. **If you frequently consult social networks, how much time do you spend on them (average per day)? (in minutes)**

**Social Networks and Covid-19**

1. **What is (are) the source(s) of information you are using to inform yourself about the current outbreak of the Covid-19 Coronavirus?**

TV news

Newspapers

Scientific literature / Research articles

Radio

Phone application dedicated to information (Google News, Huffington Post, etc.).

Social networks

Institutional letters and e-mails (crisis unit, academic societies, etc.)

Discussions with other health professional colleagues

Other

1. **If you have WhatsApp, do you use it to discuss the Covid-19 epidemic with other professionals?**

Yes

No

1. **If you have WhatsApp, do you participate with other professionals in a group dedicated exclusively to the current Covid-19 epidemic?**

Yes

No

1. **Do you work in a unit that is (or will be) involved in the management of patients with Covid-19?**

Yes

No

1. **Have you ever been directly involved in the management of a patient hospitalized for a Covid-19? (contact with patient, care, clinical examination, etc.) ***

Yes

No

1. **On a scale of 0 to 10, how informed do you think you are about the current epidemic of Covid-19? ***

Not informed at all 0 1 2 3 4 5 6 7 8 9 10 Perfectly informed

1. **On a scale of 0 to 10, how anxious do you feel about the current epidemic of Covid-19? ***

No anxiety 0 1 2 3 4 5 6 7 8 9 10 Major Anxiety

1. **If you are using WhatsApp to share information with professionals on Covid-19 epidemic, how do you evaluate its impact on your perception of the epidemic?**

Very anxious 0 1 2 3 4 5 6 7 8 9 10 Very reassuring

1. **If you are using social networks to inform yourself about the current Covid-19 epidemic, how do you estimate the impact this is having on your perception of the epidemic?**

Very anxious 0 1 2 3 4 5 6 7 8 9 10 Very reassuring
